# Understanding Weather and Hospital Admissions Patterns to Inform Climate Change Adaptation Strategies in the Healthcare Sector in Uganda

**DOI:** 10.3390/ijerph15112402

**Published:** 2018-10-29

**Authors:** Katherine E. Bishop-Williams, Lea Berrang-Ford, Jan M. Sargeant, David L. Pearl, Shuaib Lwasa, Didacus Bambaiha Namanya, Victoria L. Edge, Ashlee Cunsolo, IHACC Research Team, Bwindi Community Hospital, Yi Huang, James Ford, Patricia Garcia, Sherilee L. Harper

**Affiliations:** 1 Department of Population Medicine, University of Guelph, Guelph, ON N1G 2W1, Canada; sargeanj@uoguelph.ca (J.M.S.); dpearl@uoguelph.ca (D.L.P.); victoria.edge@canada.ca (V.L.E.); 2 Indigenous Health Adaptation to Climate Change Research Team: Cesar Carcamo, Edmonton, AB T6G 2R3, Canada; l.berrangford@leeds.ac.uk (L.B.-F.); shuaiblwasa@gmail.com (S.L.); didas.namanya@health.go.ug (D.B.N.); carlee@uoguelph.ca (I.R.T.); j.ford2@leeds.ac.uk (J.F.); patricia.garcia@upch.pe (P.G.); 3 Priestley International Centre for Climate, University of Leeds, Leeds LS2 9JT, UK; 4 Centre for Public Health and Zoonoses, University of Guelph, Guelph, ON N1G 2W1, Canada; 5 Department of Geography, Geo-Informatics and Climatic Sciences, School of Forestry, Environmental and Geographical Sciences, College of Agricultural and Environmental Sciences, Makerere University, Kampala, Uganda; 6 Ministry of Health, Kampala, Uganda; 7 Faculty of Health Sciences, Uganda Martyrs University, Kampala, Uganda; 8 Public Health Agency of Canada, Ottawa, ON K1A 019, Canada; 9 Labrador Institute, Memorial University, Happy Valley-Goose Bay, NL A0P 1E0, Canada; ashlee.cunsolo@mun.ca; 10 Bwindi Community Hospital, Kanungu District 2JJ8+GP, Uganda; patomsbwindihospital@gmail.com; 11 Department of Atmospheric and Oceanic Sciences, McGill University, Montreal, QC H3A 0G4, Canada; yi.huang@mcgill.ca; 12 Facultad de Salud Publica y Administracion, Universidad Peruana Cayetano Heredia, Lima 15102, Peru; 13 School of Public Health, University of Alberta, Edmonton, AB T6G 2R3, Canada

**Keywords:** season, meteorological parameters, weather, temperature, precipitation, climate change, hospital admissions, hospital planning, climate change adaptation, Southwestern Uganda

## Abstract

*Background:* Season and weather are associated with many health outcomes, which can influence hospital admission rates. We examined associations between hospital admissions (all diagnoses) and local meteorological parameters in Southwestern Uganda, with the aim of supporting hospital planning and preparedness in the context of climate change. *Methods*: Hospital admissions data and meteorological data were collected from Bwindi Community Hospital and a satellite database of weather conditions, respectively (2011 to 2014). Descriptive statistics were used to describe admission patterns. A mixed-effects Poisson regression model was fitted to investigate associations between hospital admissions and season, precipitation, and temperature. *Results:* Admission counts were highest for acute respiratory infections, malaria, and acute gastrointestinal illness, which are climate-sensitive diseases. Hospital admissions were 1.16 (95% CI: 1.04, 1.31; *p* = 0.008) times higher during extreme high temperatures (i.e., >95th percentile) on the day of admission. Hospital admissions association with season depended on year; admissions were higher in the dry season than the rainy season every year, except for 2014. *Discussion*: Effective adaptation strategy characteristics include being low-cost and quick and practical to implement at local scales. Herein, we illustrate how analyzing hospital data alongside meteorological parameters may inform climate-health planning in low-resource contexts.

## 1. Introduction

Counts of hospital admissions have been associated with a variety of environmental factors, including seasonal and meteorological parameters [1,2]. However, the direction and magnitude of the associations vary globally [1,3]. In the context of climate change adaptation [4], these geo-spatial variances matter, as effective climate change adaptation often requires localized and context-specific actions [5,6,7,8]. For this reason, it is important to document and understand localized associations between meteorological parameters and hospital admissions as a potential way to inform planning and preparedness measures that can support climate change adaptation in the healthcare sector [3,4,9,10].

Research investigating associations of seasonal and meteorological parameters with hospital admissions has predominantly focused on high-income regions, including North America and Europe [3,11]. Some research is available for low-income regions [1,12], including Africa [13]. Of the little research on seasonal and meteorological associations with hospital admissions that has been conducted in Africa, much has focused on specific health outcomes, which vary by host, environment, and agent. For example, in East Africa, warm temperatures were associated with increases in cholera epidemics [14] and hospital admissions for malaria [4]. In contrast, cold temperatures were associated with increases in hospital admissions for heart failure [10] and hypertension in Nigeria [10,15], and for preeclampsia in South Africa [16]. African rainy seasons have been linked to increases in hospital admissions for hypertension in Nigeria [10,15], and to pneumonia, malnutrition, and snake bites in Zambia [9]. Furthermore, the incidence of other diagnoses, such as diarrhea and malnutrition [17], and hospital admissions for respiratory syncytial virus (RSV) [18], have been associated with increased precipitation. Dry seasons have been linked to hospital admissions for some respiratory illnesses and coughing [19]. Moreover, the association of meteorological parameters with hospital admissions for a particular disease may vary by climate region [12]. For example, in Gambia, the direction of the association between temperature and RSV admissions depended on whether the region is a temperate or tropical climate, while the association between precipitation and RSV was consistent regardless of climate region [18]. Therefore, associations between disease and meteorological parameters identified in a number of African studies tend to be locally specific and vary by health outcomes, and little is known about the collective impact (i.e., overall influence) of meteorological or seasonal parameters on hospital admissions for all diagnoses.

Understanding how hospital admissions are currently associated with meteorological parameters at local scales may help the healthcare sector understand how health outcomes could be impacted under different climate change scenarios in the future [20,21], as well as inform localized climate-ready planning, such as staff and financial resource allocation in the nearer-term [6]. Climate-related planning is particularly important in East Africa, a region that has been identified as highly vulnerable to climate change [19,22,23], where climate change is anticipated to exacerbate already present health challenges in often stressed health systems [19,21,24]. As such, researchers, policy makers, and health service providers have called for regionally-specific studies to characterize how meteorological parameters and season are associated with health outcomes to support effective climate change adaptation in the healthcare sector [1,3], with evidence particularly needed to inform decision making in African countries [13,21].

While other studies examine associations of a single diagnosis with season and meteorological parameters, we intentionally focus on hospital admission counts for all diagnoses, using a case study in Uganda to explore overall primary healthcare service provision demands, and discuss our findings in the context of climate change adaptation for the healthcare sector. The goal of this study was to investigate associations of seasonal and meteorological parameters (i.e., weather data) with hospital admission counts for all diagnoses in Kanungu District, Uganda. Specifically, the objectives of this study were to: (i) Describe admissions to the hospital by diagnoses; and (ii) examine associations of hospital admissions for all diagnoses with seasonal and meteorological parameters.

## 2. Methods

### 2.1. Study Location

Uganda is located within the East African Plateau and has eight key features that influence climate: Latitude, altitude, physical shape of the lands, winds, vegetation, water bodies, and the influence of humans [25]. Generally, Uganda’s climate is warm and tropical, but high altitude regions are cooler [22]. Notable climatic changes in Uganda over the last 50 years include increases in temperature, number of hot days, and number of hot nights, as well as decreases in number of cold days [22]. Recent research demonstrated that from 1993–2016, average monthly maximum temperature in Uganda has increased 0.7 to 1.2 °C and average minimum monthly temperature has increased 1.0 to 1.1 °C [21]. There is also evidence that climate change is causing decreased annual rainfall, with small, but significant, increases in frequency and intensity of heavy rainfall events [22].

**Figure 1:**
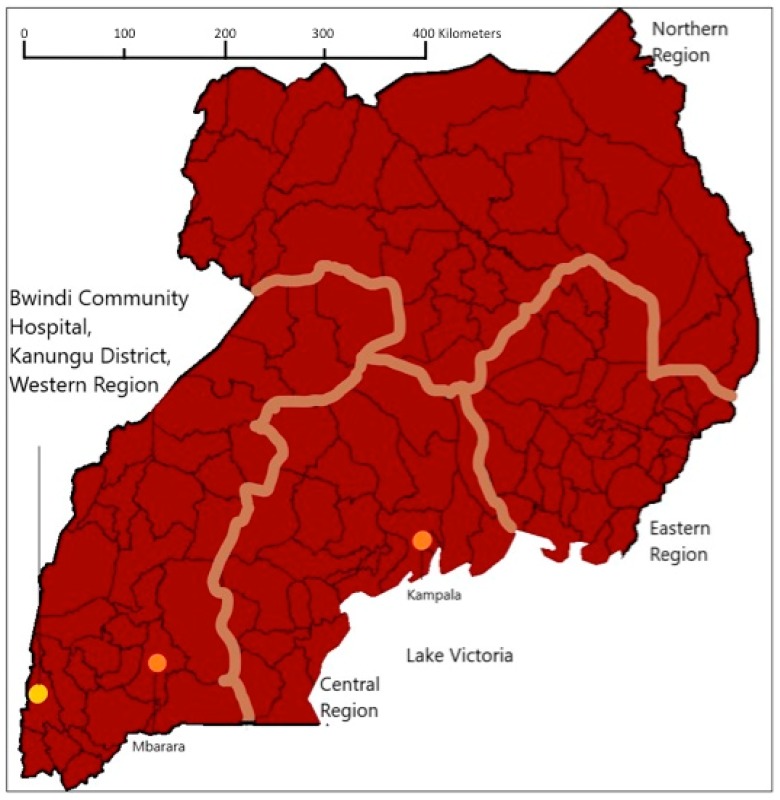

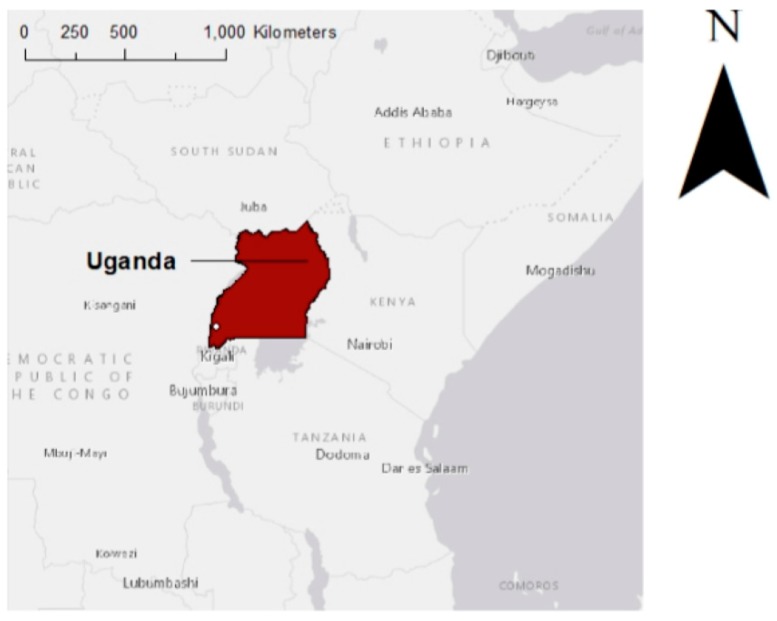
Map illustrating the location of Bwindi Community Hospital in Kanungu District, Uganda.

Kanungu District is located in the southwestern region of Uganda, bordered to the West by the Democratic Republic of the Congo, and to the North by Lake Edward, with smaller lakes, rivers, and ponds throughout (Figure 1). Kanungu District ranges from 0.44° S to 1.07° S latitude and has a relatively high altitude of approximately 1300 m [26], resulting in a highly varied geography that includes mountainous and flatter regions, as well as rain forest and savannah regions. Kanungu District has an estimated population size of 252,000 residents [27], which includes ethnic groups, such as the Bakiga [28], as well as a minority group of Indigenous Batwa [29].

Healthcare services in Kanungu District are provided by two hospitals, Bwindi Community Hospital (BCH, a private hospital) and Kambuga Hospital (a government-supported hospital [30]), and a range of health centres and small clinics. BCH is a primary healthcare facility located in Buhoma Town (Figure 1), and is a main healthcare provider for the region [19]. BCH was opened in 2003, and has 112 beds in six in-patient wards, as well as an out-patient ward. The out-patient ward treats both males and females of all ages for diagnoses not requiring overnight hospital admission. There are two in-patient wards that treat patients requiring overnight hospital admission: A pediatric ward that treats males and females 12 years of age and younger, and an adult in-patient ward that consists of separated male and female areas to treat those who are over 12 years of age. Besides the adult in-patient ward, there are maternity, surgery, and immunodeficiency wards for specific treatments of individuals over 12 years of age. The hospital operates through a fee-for-service model (i.e., privatized), with substantial donor subsidies toward an insurance scheme for residents who qualify [28]; however, many services are still cost prohibitive for some residents [31].

### 2.2. Data Collection and Management

The Indigenous Health Adaptation to Climate Change (IHACC, www.ihacc.ca) Research Team partnered with administrators at BCH to co-generate research questions, design research studies, interpret research results, and disseminate findings. EcoHealth principles guided this research process, including systems thinking, transdisciplinarity, participation, sustainability, gender and social equity, and knowledge to action [32]. The University of Guelph and McGill University Research Ethics Boards, along with BCH administrators, approved the study protocols.

To characterize hospital admission patterns in Kanungu District, clinical data for all visits to the out-patient, pediatric, and adult in-patient wards (i.e., both male and female areas of the ward) were collected from an existing electronic database at BCH. Nurses on the out-patient department recorded patient data directly into an electronic triage system, while nurses on the adult in-patient and pediatric wards recorded patient data first to paper and later entered the information into the electronic database. Out-patient records were available for 1 December 2011 to 31 December 2013; pediatric records were available for 1 January 2011 to 31 December 2014; and adult in-patient records were available for 1 December 2011 to 31 July 2014. Data collection was restricted to the period 2011 to 2014 as the hospital records prior to this period were not available in electronic formats. Paper records were used to manually fill in any gaps/missing data in the database. Data collected for each patient included diagnoses (i.e., co-morbidities), admission date, and treatment ward (i.e., out-patient, pediatric, or adult in-patient). Diagnoses at BCH were made according to the Uganda Clinical Guidelines National Guidelines for Management of Common Conditions [33].

Meteorological data were collected via satellite through a partnership with researchers at McGill University from the European Centre for Medium-Range Weather Forecasts Re-analysis (ERA)-Interim Climate Database [34], which combined data from multiple sources. The ERA-Interim climate databases have spatial resolution of 0.75° by 0.75° [34]. Daily values for total precipitation (i.e., rainfall (mm)), as well as maximum, minimum, and average temperature (°C) were obtained for all dates matching the extracted medical records (i.e., 1 January 2011 to 31 December 2014). Meteorological values extracted from the database were used to automatically generate dichotomous variables for extremely low and extremely high daily values (i.e., 5th and 95th percentiles, respectively) for precipitation; maximum, minimum, and average temperature; as well as temperature difference on a given day (i.e., difference between maximum and minimum temperature). Meteorological data were merged with clinical data for the date of admission (i.e., day zero), and lags of one to seven days were generated automatically for each meteorological variable [2]. Short lags have been recommended for this type of analysis to avoid introducing collinearity between meteorological and seasonal variables [2]. Season was categorized as rainy or dry based on date: rainy seasons were defined as March to June and September to November, and dry seasons were defined as December to February and July to August [22]. Hospital admission records were collapsed into daily counts by ward (i.e., each observation represented a count of admissions into out-patient, pediatric, or adult in-patient wards on a given date).

### 2.3. Analyses

#### 2.3.1. Descriptive Statistics

To describe admissions to the hospital by diagnoses (objective 1) and to provide context for the results overall, descriptive statistics were conducted as in other studies [1,3,12,35]. Specifically, Descriptive statistics were used to investigate BCH admission counts by ward, age group (i.e., <5 years, 6–12 years, 13–18 years, and >55 years), and sex. Counts and frequencies of admissions were tabulated. The denominator for hospital admission counts was held constant, as the population eligible to use the hospital did not change. The population in this region is not migratory and there were no other reasons for substantial changes in the population denominator from 2011–2014. Counts of diagnoses recorded more than 500 times in the database were tabulated by ward. Descriptive statistics were calculated for meteorological variables, providing further context to interpret the results.

#### 2.3.2. Regression Analyses

Two multivariable linear regressions were used to examine the effect of season and year on (1) average daily temperature, and (2) total daily precipitation. Global significance tests were used to test the overall significance of categorical variables. Two-way cross-products (i.e., interactions) were assessed between season and year. Assumptions of linear regression were assessed, including linearity of continuous independent variables, normality, and homoscedasticity.

To examine associations of hospital admissions for all diagnoses with seasonal and meteorological parameters (objective 2), regression analyses were conducted. Specifically, multilevel, multivariable Poisson regressions were used to examine if daily number of admissions for all diagnoses were associated with meteorological variables. Multilevel multivariable Poisson modeling was selected as the outcome was count data, a random effect was used to control for time, and the effect of weather on admissions was adjusted using multivariable analyses, which is similar to methods used by other climate-health research [36,37,38]. To build the multivariable models, a number of steps were followed sequentially. First, a causal diagram was created: the outcome variable was counts of hospital admissions per day, where the denominator was held constant as the number of people eligible to use the hospital (i.e., the hospital services a population of 100,000 people in Kanungu District [31]); the exposure variables of interest were meteorological parameters (i.e., four meteorological parameters and 5th and 95th percentile for each for day of admission and 7 days lag); and confounders (identified *a priori*) included season, year, and treatment ward. Confounders were forced into the models. Next, independent variables were examined using descriptive statistics. Pearson correlation coefficients were used to identify any collinearity between independent variables using a cut-point of |0.8|; if high correlations were noted between two independent variables, the variable selected for subsequent analyses was based on biological plausibility. Next, locally weighted scatter-plot smoothers were used to visually explore linearity between continuous independent variables and the outcome, and categorized if appropriate. Then, a manual forward-model building approach was used to build a multivariable Poisson regression model [35]. A series of univariable Poisson regressions were conducted to examine the unconditional association between the outcome variable and each independent variable using a liberal alpha-value (α = 0.20). All variables with *p* < 0.20 were added manually and sequentially to the model, beginning with the variable that had the lowest statistical significance *p*-value. Variables were removed if *p* > 0.05 and put back into the final model following step-wise procedures to re-examine associations before final exclusion [35]. Global significance tests were used to evaluate the statistical significance of categorical variables as a group. Interactions (i.e., cross-products) were examined based on biologically plausible combinations of independent variables. Biologically plausible interactions included interactions between meteorological parameters within one day of each other (i.e., on the same day or the day before or after) and between season and year. Autoregressive structures were investigated using graphical techniques (i.e., partial auto-correlation plots) and compared. Assessments of model fit performed on the multilevel model included examining residual plots, best linear unbiased predictor (BLUP) plots, and partial autocorrelation plots. Analyses were conducted in Stata^®^ (version 13.1, Stata Corp, College Station, TX, USA) using a standard alpha-value (α = 0.05) unless otherwise stated. In addition to multilevel Poisson models, ordinary Poisson and negative binomial models were fitted. However, based on evaluating Bayesian Information Criteria for each model, the multilevel Poisson model provided the best fit.

#### 2.3.3. Ethical Standards

The authors assert that all procedures contributing to this work comply with the ethical standards of the relevant national and institutional committees on human experimentation and with the Helsinki Declaration of 1975, as revised in 2008.

#### 2.3.4. Availability of Data

Health data used in these analyses are not available publicly. The datasets contain personal and sensitive health information for members of a small community where de-identification may be insufficient to protect the identity of participants and is not permissible according to the Research Ethics Boards at McGill University and the University of Guelph. The ethical approval number for the research is: 14-MR002 from the University of Guelph; Guelph, ON, Canada. Meteorological data from the National Centre for Atmospheric Research Climate Data Guide are available online, from https://climatedataguide.ucar.edu/.

## 3. Results

### 3.1. Description of Hospital Admissions and Meteorological Data

A total of 41,216 hospital admission records were collected from BCH between 1 January 2011 to 31 December 2014, of which 3294 were adult in-patient admissions, 5736 were pediatric in-patient admissions, and the remaining 32,186 were out-patient admissions. Females accounted for 54.6% of pediatric in-patient records and 44.0% of adult in-patient admissions; records of sex were incomplete for out-patients. The majority of patients (*n* = 29,261; 71.0%) presented with a single diagnosis, with an average of 1.3 (range: 1–14) diagnoses per hospital admission. The majority of all hospital admissions were adults (i.e., ages 19–55 years old; 41.1%), followed by children (i.e., ages 0–5 years old; 20.9%). Hospital admissions were rarely identified as a re-admission to the hospital for the same diagnosis (*n* = 87; i.e., 0.2% of cases were readmissions). Approximately half of the records for the pediatric ward and adult in-patient wards were manually entered to fill gaps in the database; however, since the out-patient department accounted for the majority of admissions, less than 10% of the total data were missing in the original database.

Admissions varied by ward, with an average of 3.0 new admissions per day on the adult in-patient ward, 3.8 new admissions per day on the pediatric ward, and 30.3 new admissions per day on the out-patient ward. Maximum daily admissions were highest on the out-patient ward (141 new admissions on a single day), lower on the pediatric ward (51 new admissions on a single day), and lowest on the adult in-patient ward (15 new admissions on a single day).

**Figure 2:**
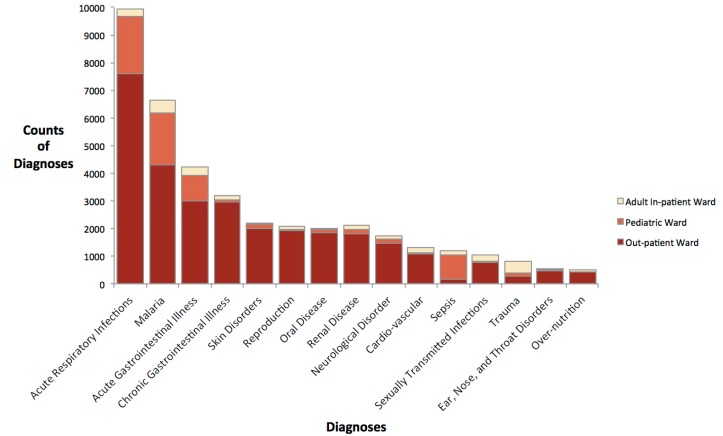
Counts of diagnoses by ward at Bwindi Community Hospital from 2011–2014 for diagnoses recorded at least 500 times in the study period.

The most common diagnoses for the out-patient ward, the pediatric ward, and for all wards combined were acute respiratory infection, followed by malaria, and acute gastrointestinal illness (Figure 2). For the adult in-patient ward, the three most common diagnoses were malaria, trauma, and acute gastrointestinal illness.

Daily average temperature ranged from 17.1 to 23.7 °C, maximum daily temperature ranged from 18.6 to 30.6 °C, while minimum daily temperature ranged from 13.2 to 19.5 °C over the four year study period. Daily temperature difference ranged from 0.0 to 14.3 °C (median: 7.8 °C). Average daily temperature increased each year from 2011 to 2014 (average daily temperature was 17.9 °C in 2011; 18.1 °C in 2012; 18.5 °C in 2013; and 18.6 °C in 2014). Daily total precipitation ranged from 0.0 to 44.8 mm (median: 1.8 mm). Average daily total rainfall varied less than 1 mm from 2011–2014 (yearly average range: 3.22 to 3.56 mm/day), with no significant trends over time.

### 3.2. Trends in Meteorological Variables

When controlling for season, the average daily temperature increased year-over-year (*p* < 0.001). Compared to 2011, the average daily temperature significantly increased by 0.16 °C in 2012 (*p* = 0.001; 95% CI: 0.06 to 0.25 °C), 0.63 °C in 2013 (*p* < 0.001; 95% CI: 0.54 to 0.73 °C), and 0.64 °C in 2014 (*p* = 0.001; 95% CI: 0.54 to 0.73 **°**C). Average daily temperature was 0.11 **°**C (*p* = 0.001, 95% CI: 0.04 to 0.18 **°**C) higher in the dry season than the wet season, after controlling for year. In a second linear regression, there was no significant change in precipitation by year (*p* = 0.188). Total daily rainfall in the dry season was, on average, 1.78 mm lower than in the wet season (*p* < 0.001, 95% CI: 1.51 to 2.05 mm).

### 3.3. Associations between Hospital Admissions and Meteorological Parameters

The best fitting model was a multilevel Poisson regression model with random effects for day and for month (Table 1). There was a significant interaction between season and year (i.e., the association between season and hospital admissions depended on year). Specifically, admissions were higher in the dry season than the rainy season, except in 2014 (Table 2). Finally, when controlling for season, year, ward, and the interaction between season and year, the rate of admissions for all diagnoses was significantly higher when the average temperature was extremely high (i.e., 95th percentile (22.3 to 29.7 °C)) on the day of admission. That is, admissions for all causes were higher on extremely warm days, contributing to an increase in average admissions from 37.1 patients per day to 43.0 patients per day.

The hospital admissions model fit the data well and the partial autocorrelation plot indicated that there was no residual temporal auto-correlation. The plot of BLUPs for month indicated they were non-parametric (i.e., not normal); however, the model fit was substantially better (i.e., lower BIC) with a random-effect for month than without, thus, the random-effect for month was maintained in the model.

## 4. Discussion

This study aimed to investigate associations of seasonal and meteorological parameters with hospital planning admissions for all diagnoses in Kanungu District, Uganda, in the context of hospital planning for climate change. Calls for policy action from the East Africa Planning for Resilience in East Africa Through Policy, Adaptation, Research, and Economic Development Project have highlighted the need for research to understand climate-sensitive diseases in this region [21]. Similar to other studies from the African continent, communicable diseases were the leading reasons for admissions at BCH [39]. The most common diagnoses at BCH (i.e., acute respiratory infections [9], acute gastrointestinal illness [40], and malaria [41]) are highly climate-sensitive diseases, and have been identified as top climate-health concerns by community members [19,28] and policy makers [21] in this region. Climate-sensitive diseases are of particular interest to inform climate adaptation for the healthcare sector [42], as they reflect the most probable, and likely most substantial, changes to the burden of illness to come. However, sufficient information on climate-sensitive diseases to inform climate change adaptation is limited, and access is often complicated in low resource regions, such as East Africa [21,43].

Climate change projections for Africa consistently project increased temperature across 121 models, showing an increase in average annual temperature ranging from 1 to 7 °C by the year 2100 [23]. Our short-term meteorological findings in Southwestern Uganda reflect this warming trend, with average annual temperature increasing by 0.6 °C, over the four year study period. In Uganda, projections indicate temperatures will continue to increase [23], which has relevance for our findings [22,23]. High temperature (i.e., above the 95th percentile) was positively associated with same-day admissions, which is consistent with research findings from other tropical African communities at high altitudes [4]. It is possible that the altitude and forest cover in this region, which have a moderating effect on temperature [25], has resulted in locally relative cold temperature adaptation of residents in this area [1,12]. Furthermore, heat related climate change adaptation strategies like air conditioning and fans [44] are not feasible or available to the majority of residents in Southwestern Uganda, which can increase admissions to hospital for a variety of causes [3]. This highlights the importance of accounting for local contextual variables in climate change adaptation, which is essential for effective anticipatory adaptation [6,43]. Local information is critical for climate change adaptation planning, since variability of processes and relationships increases at small scales [8]. This type of research can inform localized planning, such as developing and implementing early warning systems and informing planning processes, in both high-income and low-income regions, as well as contribute to the implementation of adaptation planning [45]. Supporting and improving healthcare centres is considered to be one of the most effective measures to increase climate resilience in the short-term [46].

We found significant differences in average daily temperature by year and by season, suggesting there may be variability in seasonal conditions year-to-year, which is in concordance with other regional climate analyses [21]. Specifically, we observed significant increases in temperature on a short time scale [47]. We did not observe an effect of year on precipitation in this study period; however, longer periods may show a change in precipitation patterns in the future. Since seasons were defined by historical precipitation patterns [22], the main effect of season on rainfall was expected.

We also found that admissions to the hospital were associated with an interaction (i.e., cross-product) between season and year, indicating that the effect of season on hospital admissions depends on the year in this region. As climate change progresses, seasonal variability is projected to increase [21,22,23], which could modify historic seasonal trends in disease occurrence and hospital access [21].

Climate change adaptation in the health sector in low-resource settings is often difficult, contributing to a significant adaptation deficit [43]. Numerous barriers to climate change adaptation have been identified in the Sub-Saharan African context: physical, financial, technical, social, informational, institutional and governmental, and historical, among others [43]. In response, in Uganda, successful adaptation strategies have been built on bottom-up community-based initiatives [48], had available human and financial resources to support adaptation, and had the ability to access useful and relevant information when needed [43,49,50]. In our study, we attempted to build on previous lessons learned for adaptation in Uganda by using existing datasets for hospital admissions and meteorological data, which was relatively low-cost. Furthermore, we aimed to facilitate stakeholder involvement and bidirectional communication, building on evidence that this contributes to successful adaptation [43,51]. Finally, by recognizing the importance of scale in health sector climate change adaptation [6], we focused on reaching local priorities to increase institutional support [43]. As such, investigating patterns in local meteorological parameters and hospital admissions may be beneficial for climate relevant hospital planning at local scales in low-resource contexts [6]. Understanding these context-specific relationships could be useful to improve preparedness [20], and could support shifting from reactive to anticipatory climate change adaptation for the health sector [7,52].

Some study limitations should be considered. First, this research was conducted at a local hospital, which functioned on a fee-for-service model. Thus, sampling bias should be considered with regards to vulnerable sub-populations, particularly those in the lowest income brackets [19,28], as they may be unintentionally excluded from analysis. This means that the results reflect relationships between seasonal and meteorological parameters and hospital admissions, but may not represent the relationships with disease patterns at the community-level. However, we sought to examine healthcare service provision demands to inform adaptation planning and not to describe community health status. Second, percentiles rather than thresholds for extreme temperatures were used in this research to identify local temperature extremes, which can make comparisons to disease incidence estimated in other studies difficult. By utilizing percentiles to identify locally relevant temperature extremes, we have addressed concerns from previous work that populations living in tropical climates have a higher threshold for heat tolerance [12]. Third, the data used for this research were only complete after the secondary data collection processes where researchers back-filled gaps in the electronic database from paper records. This data collection was conducted *a priori*, and thus would not have biased the results. Further, it is worth noting that the data used to backfill gaps in the electronic database were still recorded in the same way as the original records, at the same time and by the same staff members, and that only data entry into the electronic database was conducted by the research team. Fourth, this dataset represents a short time series (i.e., four years) and thus does not represent associations between hospital admissions and long-term climate or climate change parameters. Additionally, while the seasonal changes in hospital admissions at BCH could reflect disease epidemiology [9,10,15,19], it could also be explained by hospital accessibility. For instance, some factors that affect hospital access in low-income regions may change by season and/or year, including household income [17,53,54,55], road conditions [53,54,55], time availability [53], stigma [53,56], lack of available information about healthcare services [56], long wait times [53], sub-optimal interactions with healthcare providers [53], insecurity at night [57], living in rural areas [54], and/or poorly equipped facilities [57]. Fifth, while regression models, such as the mixed-effects Poisson regression used in this study, are commonly used in climate health research [3,12], these modeling strategies do not account for political and contextual factors that would further influence hospital admission patterns or future uptake of this information in Ugandan and international climate-health adaptation planning sectors. Therefore, socio-economic pathways and decision making also impact health outcomes and should be considered when discussing application of these results to inform future hospital planning in low-resource areas. Finally, while there is sufficient biological plausibility to explain an association of high temperature with hospital admissions, since we assessed a relatively large number of variables it is worth noting that our findings could also align with statistical chance, due to Type 1 (α) error, where the null hypothesis was that meteorological predictors were not associated with hospital admissions.

## 5. Conclusions

Availability and application of information for climate adaptation is limited in Uganda [43,49,50], particularly in the healthcare sector [21]. Analyses of existing healthcare use and meteorological datasets may provide additional information for hospital planning in the context of climate change in low-resource settings. Since climate change is a key concern and recognized vulnerability in Uganda, adaptation for localized environmental change and its impacts on health are crucial for healthcare providers. Healthcare planning at hospitals and health centers may be informed by an analytical tool that provides a low-cost and feasible opportunity to apply knowledge of local relationships between meteorological parameters and health while simultaneously considering common adaptation barriers in low-resource settings.

Climate-sensitive health outcomes comprise a substantial health burden [45], and thus, understanding relationships between weather and health may provide useful information for adaptive planning in localized-contexts globally. By integrating this type of information into hospital planning and climate change preparedness plans, healthcare facilities may be more equipped to deal with future influxes of patients.

## Figures and Tables

**Table 1:** Mixed-effects Poisson regression model with random-effects for month and day, outlining the associations of parameters with admissions for all causes at Bwindi Community Hospital from 2011 to 2014.

Variable ^+^		Coefficient	IRR *	*p*-Value	Confidence Interval of IRR
Season	Wet	(Ref)			
	Dry	0.567	1.764	0.21	0.225–4.290
Year	2011	(Ref)			
	2012	2.459	11.755	<0.001	5.314–26.008
	2013	2.366	10.723	<0.001	4.842–23.746
	2014	0.882	3.317	0.003	1.516–7.255
Ward	Adult In-Patient	(Ref)			
	Out-Patient	2.419	11.238	<0.001	6.634–19.037
	Pediatric	1.71	5.531	<0.001	3.250–9.412
Average Temperature on Admission Date	Below 95th percentile	(Ref)			
	Above 95th percentile	0.155	1.168	0.008	1.041–9.412
Season * Year	Wet 2011	(Ref)			
	Wet 2012	−0.474	0.622	0.445	0.184–2.104
	Wet 2013	−0.636	0.529	0.307	0.156–1.792
	Wet 2014	−1.386	0.25	0.018	0.079–0.788

^+^ A multilevel mixed-effects Poisson regression, including random effects for month and day. Standard error of the month random-effect = 0.230, standard error of the day random-effect = 0.012. Offset: Held constant as the total population eligible to use the hospital (i.e., 100,000 people). * Incidence rate ratio (IRR) calculated as the exponentiated coefficient.

**Table 2:** Table comparing hospital admission rates during (**a**) wet and dry seasons by year from 2011 to 2014, and (**b**) years 2011 to 2014 by season, at Bwindi Community Hospital.

**a. Wet and Dry Seasons by Year**				
**Year**	Wet Season IRR *	Dry Season IRR *	***p*-Value**	**Confidence Interval**
2011	REF	1.763	0.205	0.733–4.242
2012	REF	1.097	0.825	0.482–2.499
2013	REF	1.069	0.873	0.410–2.133
2014	REF	0.501	0.874	0.377–2.134
**b. Hospital Admissions Association with Years by Season**				
**Season**	**Year**	Year IRR *	***p*-Value**	**Confidence Interval**
Wet	2011	(REF)		
	2012	11.690	<0.001	5.335–25.618
	2013	10.658	<0.001	4.859–23.376
	2014	2.4152	0.030	1.090–5.354
Dry	2011	(REF)		
	2012	7.274	<0.001	2.573–14.201
	2013	5.651	<0.001	2.297–14.155
	2014	1.278	0.606	0.503–3.249

* Incidence rate ratio (IRR) calculated as the exponentiated coefficient.

## References

[1] Ma W., Xu X., Peng L., Kan H. (2011). Impact of extreme temperature on hospital admission in Shanghai, China. Sci. Total. Environ..

[2] Kinney P.L., Schwartz J., Pascal M., Petkova E., Le Tertre A., Medina S., Vautard R. (2015). Winter season mortality: Will climate warming bring benefits?. Environ. Res. Lett..

[3] Bishop-Williams K.E., Berke O., Pearl D.L., Kelton D.F. (2015). A spatial analysis of heat stress related emergency room visits in rural Southern Ontario during heat waves. BMC Emerg. Med..

[4] Githeko A.K., Ndegwa W. (2001). Predicting malaria epidemics in the Kenyan highlands using climate data: A tool for decision makers. Glob. Chang. Hum. Health.

[5] Adger W.N., Barnett J., Brown K., Marshall N., O’brien K. (2013). Cultural dimensions of climate change impacts and adaptation. Nat. Clim. Chang..

[6] Adger W.N., Arnell N.W., Tompkins E.L. (2005). Successful adaptation to climate change across scales. Glob. Environ. Chang..

[7] Heltberg R., Siegel P.B., Jorgensen S.L. (2009). Addressing human vulnerability to climate change: Toward a ‘no-regrets’ approach. Glob. Environ. Chang..

[8] Wilbanks T.J., Kates R.W. (1999). Global change in local places: How scale matters. Clim. Chang..

[9] Gernaat H., Dechering W., Voorhoeve H. (1998). Clinical epidemiology of paediatric disease at Nchelenge, north-east Zambia. Ann. Trop. Paediatr..

[10] Ansa V., Ansa V.O., Ekott J.U., Essien I.O., Bassey E.O. (2008). Seasonal variation in admission for heart failure, hypertension and stroke. Ann. Afr. Med..

[11] Whitman S., Good G., Donoghue E.R., Benbow N., Shou W., Mou S. (1997). Mortality in Chicago attributed to the July 1995 heat wave. Am. J. Public Health.

[12] Pudpong N., Hajat S. (2011). High temperature effects on out-patient visits and hospital admissions in Chiang Mai, Thailand. Sci. Total. Environ..

[13] Kudamatsu M., Persson T., Strömberg D. (2012). Weather and Infant Mortality in Africa.

[14] Olago D., Marshall M., Wandiga S.O., Opondo M., Yanda P.Z., Kangalawe R., Githeko A., Downs T., Opere A., Kabumbuli R. (2007). Climatic, socio-economic, and health factors affecting human vulnerability to cholera in the Lake Victoria basin, East Africa. AMBIO A J. Hum. Environ..

[15] Isezuo S. (2003). Seasonal variation in hospitalisation for hypertension-related morbidities in Sokoto, north-western Nigeria. Int. J. Circumpolar Health.

[16] Immink A., Scherjon S., Wolterbeek R., Wilhelm Steyn D. (2008). Seasonal influence on the admittance of pre-eclampsia patients in Tygerberg Hospital. Acta Obstet. Et Gynecol. Scand..

[17] Wang L., Kanji S., Bandyopadhyay S. (2009). The Health Impact of Extreme Weather Events in Sub-Saharan Africa.

[18] Van der Sande M.A., Goetghebuer T., Sanneh M., Whittle H.C., Weber M.W. (2004). Seasonal variation in respiratory syncytial virus epidemics in the Gambia, West Africa. Pediatr. Infect. Dis. J..

[19] Berrang-Ford L., Dingle K., Ford J.D., Lee C., Lwasa S., Namanya D.B., Henderson J., Llanos A., Carcamo C., Edge V. (2012). Vulnerability of indigenous health to climate change: A case study of Uganda’s Batwa Pygmies. Soc. Sci. Med..

[20] Ebi K.L., Kovats R.S., Menne B. (2006). An approach for assessing human health vulnerability and public health interventions to adapt to climate change. Environ. Health Perspect..

[21] United States Agency for International Development (2018). Climate Change Vulnerability and Adaptation in East Africa: Health, Sanitation, and Human Settlements. Fact Sheets.

[22] McSweeney C., New M., Lizcano G. (2010). UNDP Climate Change Country Profiles: Uganda.

[23] Boehlert B., Strzepek K.M., Groves D., Hewitson B., Jack C., Cervigni R., Liden R., Neumann J.E., Strzepek K.M. (2015). Climate Change Projections in Africa. Enhancing the Climate Resilience of Africa’s Infrastructure: The Power and Water Sectors.

[24] Ohenjo N., Willis R., Jackson D., Nettleton C., Good K., Mugarura B. (2006). Health of Indigenous people in Africa. Lancet.

[25] BakamaNume B.B. (2010). A Contemporary Geography of Uganda.

[26] Google (2016). Google Maps. www.google.ca/maps.

[27] Uganda Bureau of Statistics (2014). National Population and Housing Census 2014 Provisional Results.

[28] Labbé J., Ford J.D., Berrang-Ford L., Donnelly B., Lwasa S., Namanya D.B., Twesigomwe S., Harper S.L., IHACC Research Team (2015). Vulnerability to the health effects of climate variability in rural southwestern Uganda. Mitig. Adapt. Strat. Glob. Chang..

[29] Jackson D. (2006). The Health Situation of Women and Children in Central African Pygmy Peoples.

[30] Musinguzi G., Musinguzi G. (2006). Uganda Radio Network (URN). Kambuga Hospital to Close.

[31] Bwindi Community Hospital Patient Care. Hospital Services n.d. http://www.bwindihospital.com/patient-care.html.

[32] Charron D.F. (2012). Ecohealth Research in Practice.

[33] National Centre for Atmospheric Research (2016). Climate Data Guide. www.climatedataguide.ucar.edu.

[34] Kulldorff M., Heffernan R., Hartman J., Assunçao R., Mostashari F. (2005). A space-time permutation scan statistic for disease outbreak detection. PLoS Med..

[35] Dohoo I.R., Martin W., Stryhn H.E. (2003). Veterinary Epidemiologic Research.

[36] Liao J., Yu S., Yang F., Yang M., Hu Y., Zhang J. (2016). Short-Term Effects of Climatic Variables on Hand, Foot, and Mouth Disease in Mainland China, 2008–2013: A Multilevel Spatial Poisson Regression Model Accounting for Overdispersion. PLoS ONE.

[37] Rodrigues N.C.P., Lino V.T.S., Daumas R.P., de Noronha Andrade M.K., O’Dwyer G., Monteiro D.L.M., Gerardi A., Fernandes G.H.B.V., Ramos J.A.S., Ferreira C.E.G. (2016). Temporal and Spatial Evolution of Dengue Incidence in Brazil, 2001–2012. PLoS ONE.

[38] Mousam A., Maggioni V., Delamater P.L., Quispe A.M. (2017). Using remote sensing and modeling techniques to investigate the annual parasite incidence of malaria in Loreto, Peru. Adv. Water Resour..

[39] World Health Organization (2015). World Health Statistics 2015. World Health Statistics.

[40] Clark S., Berrang-Ford L., Lwasa S., Namanya D.B., Edge V.L., Harper S.L. (2015). IHACC Research Team. The burden and determinants of self-reported acute gastrointestinal illness in an Indigenous Batwa Pygmy population in southwestern Uganda. Epidemiol. Infect..

[41] Hamad A.A., Abd El Hamid D.N., Arnot D.E., Giha H.A., Abdel-Muhsin A.M.A., Satti G.M., Theander T.G., Creasey A.M., Babiker H.A., Elnaiem D.E.A. (2002). A marked seasonality of malaria transmsission in two rural sites in eastern Sudan. Acta Trop..

[42] Tong S., Berrang-Ford L., Lwasa S., Namanya D.B., Edge V.L., Harper S., IHACC Research Team (2016). Managing and mitigating the health risks of climate change: Calling for evidence-informed policy and action. Environ. Health Perspect..

[43] Shackleton S., Ziervogel G., Sallu S., Gill T., Tschakert P. (2015). Why is socially-just climate change adaptation in sub-Saharan Africa so challenging? A review of barriers identified from empirical cases. Wiley Interdiscip. Rev. Clim. Chang..

[44] Kovats R.S., Hajat S. (2008). Heat stress and public health: A critical review. Annu. Rev. Public Health.

[45] Watts N., Adger W.N., Agnolucci P., Blackstock J., Byass P., Cai W., Chaytor S., Colbourn T., Collins M., Cooper A. (2015). Health and climate change: Policy responses to protect public health. Lancet.

[46] Ebi K.L., Frumkin H., Hess J.J. (2017). Protecting and promoting population health in the context of climate and other global environmental changes. Anthropocene.

[47] Hartmann D., Klein Tank A., Rusticucci M., Alexander L., Broennimann S., Charabi Y.A.-R., Dentener F., Dlugokencky E., Easterling D., Kaplan A., Stocker T.F., Qin D., Plattner G.-K., Tignor M.M.B., Allen S.K., Boschung J., Nauels A., Xia Y., Bex V., Midgley P.M. (2013). Technical Summary. Climate Change 2013: The Physical Science Basis. Contribution of Working Group I to the Fifth Assessment Report of the Intergovernmental Panel on Climate Change.

[48] Ford J.D., Sherman M., Berrang-Ford L., Llanos A., Carcamo C., Harper S., Lwasa S., Namanya D., Marcello T., Maillet M. (2018). Preparing for the health impacts of climate change in Indigenous communities: The role of community-based adaptation. Glob. Environ. Chang..

[49] Ludi E., Jones L., Levine S. (2012). Changing Focus? How to Take Adaptive Capacity Seriously.

[50] Roncoli C., Orlove B.S., Kabugo M.R., Waiswa M.M. (2011). Cultural styles of participation in farmers’ discussions of seasonal climate forecasts in Uganda. Agric. Hum. Values.

[51] Ebi K.L., Semenza J.C. (2008). Community-based adaptation to the health impacts of climate change. Am. J. Prev. Med..

[52] Berrang-Ford L., Ford J.D., Paterson J. (2011). Are we adapting to climate change?. Glob. Environ. Chang..

[53] Duff P., Kipp W., Wild T.C., Rubaale T., Okech-Ojony J. (2010). Barriers to accessing highly active antiretroviral therapy by HIV-positive women attending an antenatal clinic in a regional hospital in western Uganda. J. Int. AIDS Soc..

[54] Hjortsberg C., Mwikisa C. (2002). Cost of access to health services in Zambia. Health Policy Plan..

[55] Sacks E., Vail D., Austin-Evelyn K., Greeson D., Atuyambe L.M., Macwan’gi M., Kruk M.E., Grépin K.A. (2015). Factors influencing modes of transport and travel time for obstetric care: A mixed methods study in Zambia and Uganda. Health Policy Plan..

[56] Posse M., Meheus F., Van Asten H., Van Der Ven A., Baltussen R. (2008). Barriers to access to antiretroviral treatment in developing countries: A review. Trop. Med. Int. Health.

[57] Essendi H., Mills S., Fotso J.-C. (2011). Barriers to formal emergency obstetric care services’ utilization. J. Urban Health.

